# News About Terrorism and Attitudes Toward Countries: The Role of Mortality Salience and Intergroup Threat

**DOI:** 10.11621/pir2021.0107

**Published:** 2021-06-30

**Authors:** Irina S. Prusova, Olga A. Gulevich

**Affiliations:** National Research University “Higher School of Economics”, Moscow, Russia

**Keywords:** mortality salience, intergroup threat, attitudes toward countries, Terror Management Theory, Intergroup Threat Theory

## Abstract

**Background:**

Media reports on armed fights or terror attacks introduce reminders of death into people’s daily lives. When people feel non-specific threats (mortality salience) or specific threats (intergroup threats), they may demonstrate unfavorable attitudes toward national outgroups. The issue is mostly analyzed today in line with Terror Management Theory and Intergroup Threat Theory.

**Objective:**

To examine such threats in the Russian context, and the impact of mortality salience (MS) on attitudes toward national outgroups that induced different levels of perceived intergroup threat.

**Design:**

In two studies, participants watched films and completed questionnaires about social distance, social thermometer, and trust toward “more or less threatening” countries. In Study 1, 120 Russian students were assigned to six groups via experimental design: 3 (MS: terrorist attacks in Europe, terrorist attacks in Russia, or a control group watching a video about dental treatment) x 2 (country: Ukraine and Belarus). In Study 2, 122 participants were similarly divided into six groups, evaluating attitudes toward the USA and China.

**Results:**

Study 1 showed that MS mostly increased unfavorable attitudes toward a country perceived as more threatening (Ukraine) than toward one perceived as less threatening (Belarus). Study 2 indicated the same effect on attitudes toward both more (the USA) and less (China) threatening outgroups.

**Conclusion:**

The results identified contradictory tendencies in MS effect, in line with Terror Management Theory and Intergroup Threat Theory. The findings could be used in improving relationships from an international perspective.

## Introduction

The mass media influence our perception of the world in the context of social meanings (Altheide, 2007). Today, in “the culture of fear” (wars, conflicts, terror attacks), researchers have found a significant role for correspondent discourse in the media via expectation of danger and symbolic awareness of threat in everyday life (Skoll, 2016). The term “terrorism” is frequently identified as a “threat to group existence”, a “threat to one’s way of life”, an “ideological instrument of control”, or a “form of communication” (Skoll, 2016; Skoll & Korstanje, 2013). People perceive terrorist attacks in exaggerated ways around the world that are mostly covered in the media (Howie, 2012). News about terrorism includes common aspects, e.g., a bloody incident, information about the terrorist organization (targets, portraits), number of victims, counterter-rorism politics, and global outcomes of terrorism (Skoll, 2016; Skoll, & Korstanje, 2013). News about terrorist attacks might increase support for military policies and unfavorable attitudes toward groups associated with terrorist “agenda” and frames (e.g., support of Iraq War, Syrian conflict) (Das, Bushman, Bezemer, Kerkhof, & Vermeulen, 2009; Echebarria-Echabe & Fernández-Guede, 2006; Korstanje, 2019).

The effect of terrorism news might be linked with a higher level of threat through reminders of death (non-specific threat) and perceived threat from a particular outgroup (specific threat) (Das et al., 2009; Jang, 2019; Pan, Zhou, & Hayes, 2017). People encounter a specific threat in the case of actual danger from a particular outgroup in the realistic or symbolic perspectives that, as a consequence, impact their attitudes toward this community (Stephan, Ybarra, & Rios, 2015). Pan, Zhou, and Hayes (2017) found that TV news with death-related content activated negative judgments on the immigration issue when immigrant perpetrators were negatively illustrated in the news.

A non-specific threat appears as a result of situational stimuli (e.g., information about terror attacks or pictures with destroyed buildings) and also precedes the evaluation of different objects through thoughts about mortality or about an unpredictable end of life (Jang, 2019; Landau et al., 2004; Vail, Arndt, Motyl, & Pyszczynski, 2012). For instance, death reminders from terrorism news in Western countries increased unfavorable attitudes toward Islamic migrants (Ahmad, 2015; Landau et al., 2004).

In numerous studies, researchers have examined the effect of terrorism news on specific groups portrayed in the news content as potentially “evil” without analysis of neutral groups (Das, Bushman, Bezemer, Kerkhof, & Vermeulen, 2009). For example, after 9/11, researchers in the USA frequently analyzed different forms of discrimination against Arabs and Muslims (Ahmad, 2015; Das et al., 2009; Landau et al., 2004). Terrorism news might also increase unfavorable attitudes toward any outgroup, regardless of news content or the viewer’s socio-cultural background, when the news reports confront consumers with their mortality. For instance, news about 9/11 increased discrimination not only among Europeans toward Arabs but also among Arabs toward Europeans. Therefore, the research question is whether terrorism news influences attitudes toward any outgroups or only those that appear threatening.

The term “existential threat” as used in the psychological literature has the central meaning “perceived threat to survival” (from physical annihilation to a threat to one’s way of life) in personal and social perspectives (Hirschberger, Ein-Dor, Leidner, & Saguy, 2016). Notably, the perception of an existential threat may not be linked with objective causes. Some studies conceptualized the existential threat to be rooted in the personal fear of death (Pyszczynski, Solomon, & Greenberg, 2003), whereas others defined it as a collective-level threat to the in-group’s existence (Riek, Mania, & Gaertner, 2006; Wohl & Branscombe, 2009).

To define these types of threat, researchers suggest two psychological theories, Intergroup Threat Theory (ITT) (Stephan, Ybarra, & Rios, 2015) and Terror Management Theory (TMT) (Greenberg, Pyszczynski, & Solomon, 1986). The ITT defines specific perceived threats as a basic cause of attitudes toward particular outgroups, whereas the TMT focuses on non-specific threats that worsen attitudes toward different groups. Previous studies have shown that both specific and non-specific threats independently influence attitudes toward outgroups (Burke, Martens, & Faucher, 2010; Stephan et al., 2015). To shed light on the relationships between these types of threat, the goal of the current study was to examine the effect of mortality salience (MS) on attitudes toward outgroups inducing different levels of intergroup threat.

### Mortality Salience as a Source of Perceived Threat

Terror Management Theory posits that people have an instinct of self-preservation and awareness of the inevitability of death (Solomon, Greenberg, & Pyszczynski, 1991). A wide range of similar adaptive mechanisms is represented in plenty of animal species, but only humans have the ability for abstract thinking that enables them to grasp the inevitability of death (Solomon, Greenberg, & Pyszczynski, 1991). The contradiction between the instinct of self-preservation and the inevitability of death causes “paralyzing fear” (Solomon et al., 1991). It has been suggested that dealing with this fear is a dual process. The current model indicates a sequential process of proximal and distal defenses following an encounter with death-related information (Greenberg, Arndt, Simon, Pyszczynski, & Solomon, 2000). Proximal defense is activated to remove the death-related thoughts from conscious awareness and reduce the negative affect (Greenberg et al., 2000). After proximal suppression, distal defense actualizes a cultural anxiety buffer including self-esteem (beliefs that one’s behavior corresponds to certain cultural values and standards) and one’s cultural worldview (an idea of reality that indicates the meaning, purpose, and importance of human life, standards by which human behavior can be assessed, and hope that adherence to these rules and values will empower people to be immortalized) (Pyszczynski, Solomon, & Greenberg, 2015).

To verify the correctness of a worldview, a person has to focus on the attitudes of others: either an individual or a social group. Those who share a person’s worldview increase that person’s confidence in its accuracy, while those with different positions reduce this assurance. Thus, people tend to express more positive attitudes toward others who confirm their views and are more aggressive toward dissimilar others. It has been found that reminders of death increase this tendency (Burke et al., 2010). Specifically, psychological studies have shown that if reminded about death, people express more negative attitudes toward sexual, religious, racial, ethnic, and national outgroups (Greenberg & Kosloff , 2008). For instance, White participants express more negative attitudes toward African-Americans (Bradley, Kennison, Burke, & Chaney, 2012); students at American university toward Jews (Cohen, Harber, Jussim, & Bhasin, 2009); Israeli participants toward Iran (Hirschberger & Ein-Dor, 2006); Scots toward the English (Castano, 2004); Germans toward Turks (Pyszczynski, Solomon, & Greenberg, 2003); Italians toward Germans (Castano, Yzerbyt, Paladino, & Sacchi, 2002); Europeans toward Arabs and migrants (Das,et. al., 2009; Pan et al., 2017); Arabs toward Europeans (Das et al., 2009); and Nigerian students toward the Hausa people (Ezeh, Mefoh, Nwonyi, & Aliche, 2017).

Previous studies of TMT analyzed the role of pre-existing beliefs in the context of intergroup relations (e.g., political conservatism, right-wing authoritarianism, social dominance orientation, values). For example, Greenberg, Simon, Pyszczynski, Solomon, and Chatel (1992) found that only conservatives under MS conditions showed more unfavorable attitudes toward others. Similarly, right-wing Israelis demonstrated support for militarism against those they believe “violate” their worldview (Hirschberger & Ein-Dor, 2006). At the same time, representation of human values under the MS condition led to reduction of anti-Arab prejudice (Motyl et al., 2011). When TMT did not consider the pre-existing attitudes toward outgroups, there is empirical support to expect a general “conservative shift” (reminders of death intensified intergroup hostility) (Jost, 2019). This contradiction raised questions about the role of cultural worldview in Terror Management Theory and System Justification Theory. In this study, we considered the pre-existing level of perceived intergroup threat from different countries.

Researchers in the TMT framework also focus on the personal death condition without detailed analysis of collective threats and consider these threats interchangeable (Hirschberger et al., 2016). At the same time, TMT posits that collective identity might reduce the paralyzing fear of death (Pyszczynski et al., 2015), although the threat of death can intensify death-related thoughts (Schimel, Hayes, Williams, & Jahrig, 2007). In the Russian context, people avoid direct questions about personal death that researchers have frequently used in experimental procedures (e.g., Levada-Center, 2018). Therefore, in the current study we focused on terrorism news (without information about potential “evil”) as the mortality salience manipulation.

TMT also indicates the significant role of cognitive defense mechanisms in mediating the impact of terrorism news on outgroup prejudice that has been difficult to examine and interpret (e.g., Das et al. 2009). In previous studies, researchers did not control the activation of death fear in an experimental procedure that induced additional questions about MS manipulation (Burke et al. 2010). An additional scale of the fear of death might actualize the mortality salience effect in the control group (Pyszczynski et al., 2015). Therefore, in the current research, we tried to control the experimental manipulation (terrorism news) through a word-completion task and the level of negative affect.

### Interaction Between Mortality Salience and Intergroup Threat

Intergroup Threat Theory proposes that perception of outgroups through realistic or symbolic threats determines attitudes toward them (Stephan et al., 2015). A perceived realistic threat is linked to physical and material harm; this type of threat is more likely than a perceived symbolic threat to induce prejudices against perceived dangerous and competitive outgroups (Stephan et al., 2015). People who perceive a realistic threat believe that outgroup members might harm the ingroup’s resources or economic welfare. For instance, the 9/11 teerrorist attack intensified the economic crisis in 2001 and as a result fostered a perception of a realistic threat from Mexican immigrants (Hitlan, Carrillo, Zárate, & Aikman, 2007). A perceived symbolic threat is associated with changes in values and way of life (Stephan et al., 2015). People who perceived a symbolic threat assumed that outgroup members might introduce different cultural standards and destroy the group’s way of life. Stephan, Renfro, Esses, Stephan, and Martin (2005) say that both types of threat increase unfavorable attitudes toward outgroups. For instance, Americans who encountered information about Rwandan immigrants that posed both realistic and symbolic threats expressed more unfavorable attitudes toward those immigrants (Stephan et al., 2005).

The effect of perceived intergroup threat may increase the impact of mortality salience on attitudes toward national outgroups. Previous studies have shown that after reminders of death, American students demonstrated more negative attitudes toward Israel but not to India (Cohen et al., 2009), and toward illegal aliens from Mexico City but not from Vancouver (Bassett & Connelly, 2011). This difference might be linked with pre-existing views toward the mentioned groups; in other words, the participants might have previously perceived citizens of India and Canada as less threatening than those of Israel and Mexico; however, the researchers did not pretest the target groups. Therefore, in terms of the terror management perspective, we proposed that reminders of death will mostly reinforce unfavorable attitudes toward more threatening outgroups (**research hypothesis**).

### Overview of Research

The present research integrates findings from Terror Management Theory and Intergroup Threat Theory to shed light on the MS effect on attitudes toward outgroups. The goal of the two experimental studies was to determine whether reminders of death cause unfavorable attitudes toward countries perceived as more threatening, in comparison with less threatening ones. In the experimental design we focused on two factors, mortality salience and perceived threat (less and more threatening countries). Moreover, national outgroups might be considered not only with respect to intergroup threat, but also to intergroup similarity. Intergroup similarity is the extent to which individuals consider the outgroup as similar to their own (Stephan et al., 2005). In previous studies, researchers did not examine the objective or perceived similarity as additional factors. In the present study, we tried to take into account the objective similarity (e.g., traditions, language, roots) and conducted two independent studies, with an analysis of attitudes toward culturally similar and dissimilar countries.

To actualize reminders of death, we focused on terrorism news reports. The specifics of news with death-related content might illustrate different effects on attitudes toward national outgroups. Interestingly, Perloff (2016) assumed that territorially close threats caused greater prejudice against “national outgroups” than distant threats. Das and colleagues (2009) found that news about terrorist attacks in the USA did not increase prejudices against Muslims among Dutch study participants, but news about crime in the Netherlands led to a negative view of Arabs as a national outgroup. However, in previous studies, researchers did not experimentally compare the effect of physically close and distant threats on attitudes toward countries. To examine the difference between close and distant threats, we reminded participants about close threats (news about terrorist attacks in Russia) or distant threats (news about terrorist attacks in Europe). The content of the video did not include any information about the countries that were the targets of the participants’ attitudes. After experimental manipulation, participants expressed their attitudes toward countries that might be distinguished by the level of intergroup threat in different cultural perspectives (similar and dissimilar). In both studies, we proposed to evaluate attitudes toward two national groups (more and less threatening), relations with which have been discussed in the Russian media.

Study 1 tested our hypothesis in a culturally similar context, making use of countries with whom Russia has had similar values, traditions, and language (the East Slavic language group), and a long history of co-existence within a common state, the Soviet Union (Ukraine and Belarus). Following the collapse of the Soviet Union, relations between Russia and the other two countries developed in different ways. According to a survey conducted by Levada-Center, Belarus ranked first on the list of friendly countries: 46% of respondents considered Belarus as a friend of Russia (Levada-Center, 2017), whereas Ukraine ranked second on the list of unfriendly countries: 50% of respondents considered Ukraine an enemy of Russia. Thus, these countries were considered culturally similar to Russia, but were differentiated in the level of perceived threat (Belarus–friend, Ukraine–enemy).

Study 2 examined attitudes toward culturally dissimilar countries: China and the USA. According to a Levada-Center study (2017), China ranked second on the list of friendly countries: 39% of respondents considered China a friend of Russia; the USA ranked first on the list of unfriendly countries: 69% of respondents considered the USA an enemy of Russia. Thus, these countries were considered culturally dissimilar to Russia, but differentiated in the level of threat (China–friend, USA–enemy).

## STUDY 1

### Methods

#### Participants

Participants were 120 students (76 women; ages 18-30, M = 21.06, SD = 2.28) from different departments of the Higher School of Economics (HSE) and the State Academic University for the Humanities (SAUH), who participated in exchange for course credits. The number of students from the different universities was the same in the different experimental conditions. The data were collected from November 2017 to February 2018. Participants were randomly assigned to one of the conditions of a between-group design: 3 (mortality salience: terrorist attacks in Europe, terrorist attacks in Russia, or dental treatment) x 2 (type of outgroup: more or less threatening countries).

To determine the required number of participants in our studies, we conducted an a priori power analysis. In line with Burke, Martens, and Fancher’s (2013) meta-analysis related to the MS effect on cultural worldview and self-esteem, the mean effect size was 0.35. We transformed the current r-value into the f-value (0.37), using Psychometrica software to analyze the relevant number of participants corresponding to our experimental design and specifics of data analysis (MANOVA) (Psychometrica, 2019). A priori power analysis by G*Power (Faul, Erdfelder, Buchner, & Lang, 2009) showed that a sample size of 102 (with around 17 participants in each experimental condition) was appropriate to represent the previously mentioned effect-size (F = .0.37) with sufficient power (1 – ß > .80).

We chose a student sample for two reasons: (1) previous studies showed that MS effects were significantly higher for college students than for other participants (Burke et al., 2010); and (2) the availability of additional motivation via bonuses and similar conditions.

#### Procedure

Participants were asked to take part in a study on individual worldviews that consisted of two parts. The first part was hosted on the Google website and was represented as a study about how different countries are perceived by Russian citizens. Participants were randomly assigned to evaluate the perceived threat from either Belarus (N = 60) or Ukraine (N = 60). At the end of the first session, they received an identification number for the second session.

The second session was held a week after the first and included a face-to-face procedure that lasted about 30 minutes. After giving their informed consent, participants watched one of the three videos (terror attacks in Europe, terror attacks in Russia, or dental treatment), answered questions about it, and completed control measures to test the MS manipulation. The dependent variables were participants’ attitudes toward Belarussians or Ukrainians (feeling thermometer, group trust, and social distance). Participants were required to evaluate the same country in both the first and second sessions. Finally, participants were given a debriefing, in which they received information about the actual aim of the research and watched a cartoon intended to induce positive emotions.

##### Pretesting of target countries

To determine how these countries were perceived by the participants in our study, we used a questionnaire about perceived intergroup threat. This measure included two items about perceptions of realistic threat (“[Name of country]’s military development poses a threat to the interests of Russia”, and “[Name of country]’s economic development poses a threat to the economy of Russia”), and two items about symbolic threats (“[Name of country] is a threat to Russian culture”, and “[Name of country] is a threat to Russian values and norms”) (Stephan, Ybarra, Martinez, Schwarzwald, & Tur-Kaspa, 1998). The analysis showed that all statements formed one scale of “intergroup threat” (a = .91). Participants might agree or disagree with statements about Ukraine and Belarus along a 5-point scale (from 1 = *completely disagree* to 5 = *completely agree*).

To test the perceived threats from Ukraine and Belarus, we used the independent t-test. The results showed that participants perceived a greater threat (*t*(118) = -5.81, *p* < .001, Cohen’s *d* = 1.057) from Ukraine (M = 2.84, SD = 1.19) than from Belarus (M = 1.76, SD = .82).

##### Mortality salience manipulation

In previous studies, MS has usually been manipulated by two open-ended questions concerning death (Rosenblatt, Greenberg, Solomon, Pyszczynski, & Lyon, 1989). We used a modified version of the MS procedure, in which respondents watched a video about death or a frightening event, and then imagined themselves in those situations. The changes in our procedure were because of specifics of the Russian context: in brief, people try to avoid direct questions about death (Levada-Center, 2018).

In the current study, MS was manipulated by videos of terrorist attacks in Europe (experimental group 1)/terrorist attacks in Russia (experimental group 2), or dental treatment (control condition). In the first experimental group, participants watched video reportage of terrorist attacks in Spain, France, Belgium, and the UK, which occurred between 2015 and 2017. In the second experimental group, participants watched reportage of terrorist attacks in four Russian cities, which occurred between 2004 and 2017. In the dental treatment (control) condition, participants watched video reportage of dental treatment that illustrated caries therapy and dental implantations that had negative consequences.

Each stimulus consisted of four videos from the news programs of Russian federal media channels and lasted 10 minutes. To remind participants about their own death, we asked them to imagine themselves as a victim of terrorist attacks or as a patient at a dental clinic and to describe their thoughts and feelings.

Following the MS manipulation, all participants completed a 20-item Russian version of the Positive and Negative Affect Schedule (PANAS), which evaluated both negative (10) and positive (10) emotions (Osin, 2012; Watson, Clark, & Tellegen, 1988). Participants identified the extent to which they were immediately feeling these emotions on a scale of 1 (*very slightly*) to 5 (*extremely*). To test the effect of experimental manipulations, we used only the subscale of negative emotions.

Participants then completed a set of 10-word fragments to indicate death-related thoughts (Greenberg, Pyszczynski, Solomon, Simon, & Breus, 1994). Five of these fragments might be completed with either a death-related or a neutral word (e.g., *grob — grib* [*coffin — mushroom*]), whereas the other five fragments could be completed only in one way, resulting in words unrelated to death (e.g., *igra [play*]). The number of death-related target words was regarded as the index of MS actualization.

##### Dependent measures

Three indicators — outgroup liking, outgroup trust, and readiness to interact — were used to measure attitudes toward national outgroups.

##### Feeling thermometer

To measure liking, participants had to indicate their feelings toward Belarusians or Ukrainians on a 100-point scale (0 = *cold,* 100 = *warm*) (Nelson, 2008).

##### Group trust

Participants rated their level of trust toward Belarusians or Ukrainians (1 = *totally distrust,* 10 = *totally trust)*.

##### Social distance

To measure readiness to interact, participants reported their desire to communicate with Belarusians and Ukrainians as a family member, friend, boss, subordinate, and neighbor (Gulevich, Sarieva, & Prusova, 2015). The scale included five (Cronbach’s a = .92) 5-point Likert-scale items with 1 = *completely disagree* and 5 = *completely agree,* lower scores indicating greater social distance and higher scores lower social distance.

### Results

#### Manipulation Check

The Kruskal-Wallis test showed that the manipulation worked. Participants in all conditions demonstrated the same level of negative affect (M_exp1_ = 2.19, M_exp2_ = 2.43, M_control_ = 2.20, H = .289, *p* = .865). However, they chose more death-related words in the MS conditions than in the one for dental treatment (M_exp1_ = 2.18, M_exp2_ = 2.58, M_control_ = 1.27, H = 23.374, *p* < .001). The distant and close MS conditions did not differ from one another.

#### Main Results

To analyze the data, we used the multivariate linear model (SPSS 23.0.0). The results are shown in *Table 1*.

**Table 1:** The effect of mortality salience and type of outgroup on attitudes toward Ukraine and Belarus

IV	DV	SS	df	MS	F	p	h^2^
Mortality salience	Social thermometer	32,46.05	2	1,623.03	3.50	.033	.058
	Group trust	3.02	2	1.51	.24	.784	.004
	Social distance	1.37	2	.68	.71	.495	.012
Type of outgroup	Social thermometer	19,304.03	1	19,304.03	41.63	<.001	.268
	Group trust	110.21	1	110.22	17.79	<.001	.135
	Social distance	4.96	1	4.96	5.13	.025	.043
Mortality salience × type of outgroup	Social thermometer	6,212.02	2	3,106.01	6.70	.002	.105
	Group trust	19.52	2	9.76	1.58	.211	.027
	Social distance	2.57	2	1.29	1.33	.269	.023
Error	Social thermometer	52,859.60	114	463.68			
	Group trust	706.05	114	6.19			
	Social distance	110.25	114	.97			

The results revealed a significant effect of type of outgroup on liking (F(1, 118) = 41.63, *p* < .001, h*^2^ =* .268), trust (F(1, 118) = 17.79, *p* < .001, h*^2^ =* .135), and readiness to interact with the outgroups (F(1, 118) = 5.13, *p* = .025, h*^2^ =* .043). Indeed, participants showed significantly more positive attitudes toward Belarusians (M_liking_ = 73.23, SD_liking_ = 22.17; M _trust_ = 6.92, SD _trust_ = 2.68; M_interaction_ = 4.22, SD_interaction_ = .86) than toward Ukrainians (M_liking_ = 47.87, SD_liking_ = 23.77; M_trust_ = 5.00, SD_trust_ = 2.28; M_interaction_ = 3.82, SD_interaction_ = 1.09).

Mortality salience influenced liking of outgroups (F(1, 118) = 3.50, *p* = .033, h*^2^ =* .058). A post hoc analysis comparing the attitudes toward Ukrainians and Belarusians between conditions revealed significantly lower liking of national outgroups after watching terrorist attacks in Europe (M = 55.15, SD = 27.31) than after seeing dental treatment (M = 67.58, SD = 23.74). However, there was no difference between the terrorist attacks in Russia (M = 58.93, SD = 26.46) and the dental treatment condition.

Finally, there was interaction between mortality salience and type of country with respect to liking of the outgroup (F(2,117) = 6.70, *p* = .002, h*^2^ =* .105). A post hoc analysis indicated that after watching terrorist attacks in Russia (M = 38.35, SD = 17.96) and in European countries (M = 40.85, SD = 21.53), participants expressed less liking only toward Ukrainians than after watching dental treatment (M = 64.40, SD = 23.08).

The findings from Study 1 supported the hypothesis that mortality salience had a greater impact on attitudes toward a more threatening country than toward a less threatening one. This might be related to specifics of the target countries, since Ukraine and Belarus are culturally similar to Russia. This closeness might influence the interaction between mortality salience and the perceived threat from the outgroup. Therefore, in Study 2 we analyzed the impact of mortality salience on attitudes toward countries that are dissimilar in cultural and historical perspectives.

## STUDY 2

### Methods

#### Participants

One hundred and twenty-two students (64 women; ages 18–30, M = 21.61, SD = 2.32) from different departments of the Higher School of Economics (HSE) and the State Academic University for the Humanities (SAUH) took part in this study, in exchange for course credits. The data were collected between December 2017 and February 2018.

#### Procedure

Participants followed a similar procedure as in Study 1, but with different target countries. In this case, participants expressed their attitudes toward two territorially and culturally distant countries, China and the USA. To test the type of outgroups, we compared participants` preliminary evaluations of the perceived threat (a = .88) from China and the USA. An independent t-test showed that participants perceived a greater threat (*t*(120) = -3.52, *p* = .001, Cohen’s *d* = .638) from the USA (M = 3.07, SD = 1.15) than from China (M = 2.38, SD = 1.01).

### Results

#### Manipulation Check

The Kruskal-Wallis test showed that the MS manipulation worked. Participants in all conditions demonstrated the same level of negative affect (M_exp1_ = 2.46, M_exp2_ = 2.38, M_control_ = 2.19, H = .859, *p* = .651). However, they chose more death-related words in the distant and close mortality salience conditions than in the one for dental treatment (M_exp1_ = 2.66, M_exp2_ = 3.05, M_control_ = .83, H = 55.694, *p* < .001). The distant and close mortality salience conditions did not differ from one another.

#### Main Results

The data were analyzed in the same way as in Study 1, through the multivariate linear model (SPSS 23.0.0). The results are shown in Table 2.

**Table 2:** The effect of mortality salience and type of outgroup on attitudes toward China and the United States

IV	DV	SS	df	MS	F	P	h^2^
Mortality salience	Social thermometer	3,841.09	2	1,920.55	5.00	.008	.079
	Group trust	72.63	2	36.31	8.44	.000	.127
	Social distance	.76	2	.38	.41	.663	.007
Type of outgroup	Social thermometer	7,745.14	1	7,745.14	20.15	<.001	.148
	Group trust	94.72	1	94.72	22.01	<.001	.159
	Social distance	.11	1	.11	.12	.727	.001
Mortality type of outgroup salience ×	Social thermometer	422.24	2	211.12	.55	.579	.009
	Group trust	4.90	2	2.45	.57	.568	.010
	Social distance	.45	2	.22	..24	.785	.004
Error	Social thermometer	44,592.72	116	384.42			
	Group trust	499.16	116	4.30			
	Social distance	106.38	116	.92			

The results indicate that the type of outgroup affected liking (F(1, 120) = 20.15, *p* < .001, h*^2^ =* .148) and trust (F(1, 120) = 22.01, *p* < .001, h*^2^ =* .159) toward Americans and Chinese. Participants showed significantly greater liking and trust toward Chinese (M_liking_ = 62.81, SD_liking_ = 19.76; M_trust_ = 5.26, SD_trust_ = 2.47) than toward Americans (M_liking_ = 47.02, SD_liking_ = 20.60; M_trust_ = 3.52, SD_trust_ = 1.86). However, there was no significant difference between the readiness to interact with Chinese (M_interaction_ = 3.55, SD_interaction_ = .84) and Americans (M_interaction_ = 3.61, SD_interaction_ = 1.05).

Mortality salience impacted liking (F(1, 118) = 3.50, *p* = .033, h*^2^ =* .058) and trust (F(1, 120) = 8.44, *p* < .001, h^2^ = .127) toward national outgroups. A post hoc analysis comparing the attitudes toward Americans and Chinese between conditions revealed significantly lower liking and trust after watching terror attacks in Europe (M_liking_ = 56.14, SD_liking_ = 21.46; M_trust_ = 4.74, SD_trust_ = 2.47) than after watching dental treatment (M_liking_ = 65.08, SD_liking_ = 25.08; M_trust_ = 5.82, SD_trust_ = 2.75). However, there was no difference between the terrorist attacks in Russia (M_liking_ = 53.42, SD_liking_ = 14.94; M_trust_ = 4.05, SD_trust_ = 1.63) and dental treatment conditions.

Finally, there was no significant interaction between mortality salience and type of country. The independent analysis of each country showed that videos of terrorist attacks in Europe influenced the liking of Chinese (F(1, 40) = 4.66, *p* = .037, h^2^ = .107; M_exp1_ = 57.95, SD_exp1_ = 18.54; M_control_ = 72.25, SD_control_ = 23.68) and trust toward both Americans (F(1, 38) = 6.10, *p* = .018, h^2^ = .138; M_exp1_ = 2.80, SD _exp1_ = 1.40; M_control_ = 4.35, SD_control_ = 2.43) and Chinese (F(1, 40) = 6.33, *p* = .016, h^2^ = .140; M_exp1_ = 4.57, SD_exp1_ = 2.06; M_control_= 6.60, SD_control_ = 3.03). Participants expressed marginally less liking and trust after terror in Europe toward more and less threatening countries.

Videos of terrorist attacks in Russia impacted liking (F(1, 40) = 5.15, *p* = .029, h^2^ = .117; M_exp2_ = 58.67, SD_exp2_ = 13.54; M_control_ = 72.25, SD_control_ = 23.68) and trust (F(1, 40) = 6.40, *p* = .016, h^2^ = .141; M_exp2_ = 4.67, SD _exp2_ = 1.71; M_control_ = 6.60, SD_control_ = 3.03) only toward Chinese. Participants expressed less liking toward Chinese after information about terror in Russia. In general, mortality salience had the same effect on attitudes toward more and less threatening groups, which contradicted the research hypothesis.

## Discussion

In this study, we analyzed the effect of the interaction between mortality salience and intergroup threat on attitudes toward national outgroups. We assumed that reminders of death would mostly influence attitudes toward outgroups perceived as more threatening in comparison to those perceived as less threatening. The results allowed us to identify some trends that appeared in attitudes toward similar (Ukraine, Belarus) and dissimilar (China, USA) countries. We found that attitudes toward different countries are influenced by the perceived intergroup threat. Participants showed less positive attitudes toward more threatening countries (Ukraine, USA) than to less threatening ones (Belarus, China), which corresponds with Intergroup Threat Theory.

The intergroup threat had a greater impact on cognitive and emotional components of the attitudes than on behavioral ones. In particular, participants expressed less liking and trust toward citizens of more threatening countries (Ukraine and the USA) than toward less threatening ones (Belarus and China). Differences in social distance were found between Belarus and Ukraine only. In the present case, liking and trust might reflect personal views, while readiness to interact with outgroups could be limited by external factors (such as the low probability of communication, language barriers, social norms, etc.).

The results showed that mortality salience influenced attitudes toward national outgroups. After reminders of death, participants expressed more unfavorable attitudes toward foreign countries than in the control condition. However, a comparison of the results of Study 1 and Study 2 revealed that the MS effect might be influenced by two factors, intergroup threat and intergroup similarity. Our results suggested that people tended to show changes in liking toward culturally similar outgroups (Ukraine), whereas in the case of dissimilar outgroups (the USA and China), we observed this tendency both for liking and trust.

Based on the results, close and distant death reminders have different impacts on attitudes toward outgroups. On the whole, the reminder of death by terrorist attacks in European countries had a greater effect on attitudes toward other countries than did terrorist attacks in Russia. In the current case, MS manipulation might activate a certain level of comparison among respondents. The information about events in their own country induced participants to think about different groups living there, whereas information about events in foreign countries activated thoughts about different nations. These thoughts influenced attitudes toward the correspondent outgroups: in the first case, these groups were members of national outgroups who lived in the country (e.g., migrants or inhabitants of other regions), and in the second case they were citizens of foreign countries.

## Conclusion

The current research identified the role of mortality salience induced by terrorism news on attitudes toward more or less threatening countries. Reminders of death mostly predicted attitudes toward groups perceived as more threatening and culturally dissimilar in the Russian socio-political context. The evidence from Study 1 (culturally similar outgroups) supports the hypothesis that MS intensified unfavorable attitudes toward more threatening outgroups, whereas in Study 2 (culturally dissimilar outgroups), this tendency was observed toward both less and more threatening outgroups. These findings shed light on the intergroup threat as a factor and indicated the specifics of the mortality salience effect. Further studies should analyze groups with different levels of intergroup threat and perceived similarity, as well as consider ideological variables (social dominance orientation and right-wing authoritarianism) and social context in the long-term perspective.

## Limitations

Our research had several limitations. First, we systematically varied only the perceived intergroup threat, without a detailed analysis of perceived similarities between groups. Our assumption about the perception of greater cultural similarity between Russia and Ukraine or Belarus than with China or the USA was not supported by the measures we used. The USA and China are culturally different countries and are considered by Russians to be members of “Western” and “Eastern” civilizations, respectively. To analyze the relative importance of perceived similarity and perceived threat, further research is needed.

Second, our research measured attitudes toward outgroups that induced a weak or medium sense of threat among respondents. We did not consider groups that would have induced a greater sense of threat. The current perspective might be explained by the type of participants, which in our research was students from Moscow universities, where participants study in an international environment, in which they might have “friendly” interactions with the target groups and consider terrorist attacks in other countries as relevant stimuli. Sociological surveys in Russia have shown that younger people and those with higher education express more positive attitudes toward other ethnic and national groups than do older people and those with lower educational levels (Levada-Center, 2015). To exclude this limitation, we need to conduct further research with respondents from other socio-demographic groups.

Third, our results suggest that mortality salience had no impact on attitudes toward Belarus, which is probably considered as a culturally similar and non-threatening country. This raises a research question for further study: whether MS influences only attitudes toward those who are perceived as a “threatening/dissimilar” outgroup, without any effect on evaluations of non-threating countries. To analyze this limitation, in the future we need to compare the effect of MS on attitudes toward counties that are similar to Belarus.

Finally, we used a modified version of the MS procedure, which included both viewing the death of others and thinking about one’s own death. In previous papers, some authors combined these types of threat (internal and external). For example, Burke and colleagues (2010) did not indicate any differences in the impact of questions about one’s own death and viewing of a video showing the death of others . However, in some cases, researchers distinguished mainly between “internal” and “external” forms of threat, specifically that the type of threat might have different effects on attitudes and behavior (Onraet, Van Hiel, Dhont, & Pattyn, 2013). To shed light on these contradictions, future studies might focus on independent analysis of MS manipulations through questions about one’s own death or the deaths of other people.
